# Dynamic waitlisted design for evaluating a randomized trial of evidence-based quality improvement of comprehensive women’s health care implementation in low-performing VA facilities

**DOI:** 10.1186/s43058-020-00038-0

**Published:** 2020-06-30

**Authors:** Alison B. Hamilton, Tanya T. Olmos-Ochoa, Ismelda Canelo, Danielle Rose, Katherine J. Hoggatt, Claire Than, Elizabeth M. Yano

**Affiliations:** 1 VA HSR&D Center for the Study of Healthcare Innovation, Implementation and Policy, VA Greater Los Angeles Healthcare System, Los Angeles, CA 90073 USA; 2 Department of Psychiatry and Biobehavioral Sciences, David Geffen School of Medicine, University of California Los Angeles, Los Angeles, CA 90095 USA; 3 Research Service, San Francisco VA Health Care System, San Francisco, CA 94121 USA; 4 Department of Medicine, University of California San Francisco, San Francisco, CA 94115 USA; 5 Department of Health Policy and Management, Fielding School of Public Health, University of California Los Angeles, Los Angeles, CA 90095 USA; 6 Department of Medicine, Geffen School of Medicine, University of California Los Angeles, Los Angeles, CA 90095 USA

**Keywords:** Implementation, Evidence-based quality improvement, Women’s health, Veterans

## Abstract

**Background:**

Women’s Health Services (WHS) in the Veterans Health Administration (VA) has long partnered with VA researchers to evaluate how VA care is organized for women veterans. This partnership has yielded substantial evidence of (1) variations in women veterans’ access to comprehensive healthcare services that contribute to disparities in quality and patient experience and (2) the positive impacts of gender-specific care models for women veterans’ quality and satisfaction. In an effort to provide support specifically to sites that were low-performing in women’s health, WHS and the VA Quality Enhancement Research Initiative co-funded an effort to roll out and evaluate evidence-based quality improvement (EBQI), an implementation strategy with demonstrated effectiveness in a prior cluster randomized trial in women’s health clinics.

**Methods:**

We will identify 21 low-performing VA facilities through a combination of practice data, VA quality metrics (by gender), and other indicators. In partnership with WHS, an EBQI contractor will deliver the EBQI “package”—local consensus development and priority setting using stakeholder panels, multilevel stakeholder engagement, practice facilitation, local EBQI team training, and formative feedback—to participating sites. We propose a dynamic wait-listed design to evaluate the WHS plans for seven EBQI launches per year over 3 years. The goal is to evaluate (1) barriers and facilitators to achieving delivery of comprehensive women’s health care in low-performing VA facilities; (2) effectiveness of EBQI in supporting low-performing VA facilities to achieve improved practice features (e.g., level of comprehensive services available, care coordination arrangements, Patient Aligned Care Team (PACT) features implemented, environment of care improvements), provider/staff attitudes (e.g., improved gender awareness, women’s health knowledge and practice), quality of care, and patient experience; and (3) contextual factors, local implementation processes, and organizational changes over time.

**Discussion:**

Access to comprehensive women’s health care reduces fragmentation of care, improves patient satisfaction, and results in better patient outcomes. We hypothesize that EBQI implementation will result in changes in leadership awareness and buy-in, multilevel engagement in problem-solving, an enhanced culture of quality improvement, structural changes in care, improved provider/staff attitudes, and better quality and patient experience.

**Trial registration:**

ClinicalTrials.gov, NCT03238417. Registered 3 August 2017. Retrospectively registered, https://clinicaltrials.gov/ct2/show/study/NCT03238417

Contributions to the literature

- Evidence-based quality improvement (EQBI) is an implementation strategy that engages multilevel key stakeholders to build consensus around QI goals and trains and engages local EBQI teams to guide change efforts.
- Our study evaluates the effectiveness of using EBQI to support low-performing VA facilities in implementing comprehensive women’s health care by identifying and working through the contextual and organizational barriers and facilitators to implementation.
- Evaluation results may help inform future use of EBQI in low-performing healthcare settings.

## Background

Historically plagued by gaps in safety and privacy for women in Veterans Health Administration (VA) facilities originally designed for men, with a workforce with inconsistent and/or infrequent exposure to women patients, the VA has faced significant challenges in meeting women veterans’ complex care needs [1–3]. Ensuring access to gender-specific care and a full complement of reproductive and gynecologic health services has also contributed to higher rates of community referrals among women veterans, improving access but further fragmenting their care [4–6]. These and other challenges have led to persistent gender disparities in VA care quality and patient experience [7, 8].

Over a decade ago, VA stood up a handful of comprehensive women’s health centers in response to Government Accounting Office findings of gaps in women veterans’ care, and subsequent legislation [9]. Establishment of women’s clinics grew eightfold over the next decade [10]. However, only a fraction delivered comprehensive services like the original model programs, many focusing on gender-specific exams to help increase VA breast and cervical cancer screening rates [11]. Nonetheless, adoption of women’s health clinic models was associated with higher preventive practices and higher ratings of access, continuity, coordination, and satisfaction among women veterans [12, 13].

Multiple initiatives have also been launched to change VA culture to be more gender-sensitive, set minimum standards for training and proficiency of providers designated to see women patients, and delineate features of acceptable primary care (PC) clinic models that integrate gender-specific and mental health services in “one-stop shopping” approaches. VA Women’s Health Services (WHS) has led these efforts and set forth VA policy on “Health Care Services for Women Veterans” (VHA Handbook 1330.01, May 2010) [14], which sought to systematically improve their access to comprehensive healthcare services delivered by proficient providers and staff in environments that ensure their safety, security, and dignity. In 2010, WHS launched a mandatory annual Women’s Assessment Tool for Comprehensive Health (WATCH) to evaluate Handbook implementation, and an external evaluation comprised of site visits to over 100% of VA medical centers (VAMCs). Together, these evaluation activities have documented substantial progress and informed strategic planning and decision-making in terms of policies and resources needed to improve VA women’s health programs nationwide.

The evaluations also found that traditional top-down policy implementation—even when leveraged by evaluation feedback and multilevel women’s health champions from the local clinic, regional, and national levels—has not been uniformly successful in achieving the tenets of VA policy on improving availability of comprehensive women’s health care. Using WATCH data, WHS began to identify consistently low-performing VA facilities that would benefit from more focused organizational interventions. Based on previous success using evidence-based quality improvement (EBQI) as an implementation strategy [15, 16], WHS began rolling out EBQI in low-performing VA facilities starting in fiscal year (FY) 2017. WHS’s EBQI approach is being implemented by a VA-approved contractor, building directly on the bundle of activities tested in a previous study [15], which itself was based on EBQI efforts in previous VA randomized trials. EBQI is a systematic approach to developing research-clinical partnerships to produce tailored, evidence-based care models or redesigns [17]. EBQI activities include strategic planning designed to achieve consensus on QI targets, multilevel stakeholder engagement, external practice facilitation, local EBQI team training, and formative feedback, for which technical specifications have already been developed.

## Research aims

We propose to evaluate the effectiveness of EBQI on achievement of comprehensive women’s health care in low-performing VA facilities.

Our aims are as follows:

1. To evaluate barriers and facilitators to achieving delivery of comprehensive women’s health care in the identified low-performing VA facilities;
2. To evaluate the effectiveness of EBQI in supporting low-performing VA facilities to achieve improved:
  - Organizational features (e.g., level of comprehensive services available, care coordination arrangements, implementation of Patient Aligned Care Team (PACT) features^1^, environment of care improvements);
  - Provider/staff attitudes (e.g., improved gender awareness, women’s health knowledge and practice);
  - Quality of care and patient experience among women veteran patients; and,
3. To evaluate contextual factors, local implementation processes, and organizational changes in the participating facilities over time.

Evaluation results will inform strategies for optimizing future policy deployment and multilevel engagement efforts with the field, while also informing best practice diffusion. The focus on low-performing VAs will offer new insights, as these less studied facilities may require uniquely concentrated and/or tailored efforts.

## Methods

### Trial design

**Fig. 1:**
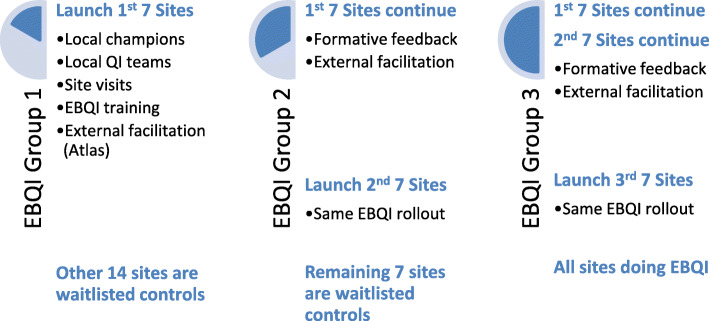
Convergent parallel mixed-methods evaluation of EBQI implementation of comprehensive women’s health care using a dynamic wait-listed design

This study is designed as a convergent parallel mixed methods evaluation [18, 19] in the context of a dynamic wait-listed design [20, 21] to evaluate the effectiveness of EBQI implementation on achievement of comprehensive women’s health care in low-performing VA facilities (Fig. 1; see CONSORT 2010 checklist) After identifying low-performing VA facilities (see the “Site selection” section), seven facilities will be randomly assigned to EBQI in the first year, holding the other 14 facilities as controls. Then another seven facilities from the wait-listed controls will be randomly assigned to EBQI in the second year, holding the remaining seven facilities as controls. In the third year, the last seven facilities will receive EBQI. No site stratification or matching criteria will be used. The study biostatistician will use www.randomization.com to assign the first seven VAMCs to EBQI or control in the first year, and then randomly assign another seven VAMCs to EBQI in the second year. The remaining seven VAMCs will receive EBQI in the third year. The EBQI vendor will enroll and launch EBQI for each facility over time based on randomization results.

### Site selection

WHS will oversee identification of eligible low-performing VA facilities using a combination of VA quality metrics (VA chart-based quality indicators), compliance with VA guidance on delivery of healthcare services to women veterans (organizational survey data), and assessments of the quality of local women’s health programs on the basis of site visits. The VA quality metrics will be obtained from VA’s performance measurement and reporting office and include presence of a gender disparity and/or national disparity for blood sugar control (HbA1c) among diabetics, annual depression screening, colorectal cancer screening, and influenza immunizations. Structural assessment data will be drawn from WATCH to rank order VA facilities; measures will include presence of local women veteran health committees, a written strategic plan for the women’s health program, a Women Veteran Program Manager, a Women’s Health Medical Director (or Women’s Health Champion in smaller facilities), a mammography coordinator, and a maternity care coordinator. Site visit data over the previous 4 years will be used to assess site ranking (between 1 and 140) based on a series of women’s health program components, the percent of women veterans assigned to a designated women’s health provider, and the percent of women veterans waiting more than 30 days for a comprehensive women’s health care appointment. WHS and a contracted support vendor will use the data to identify 30 VA facilities with the most sub-par metrics. The evaluation team will randomly sample 24 sites, providing us with three backup sites if a site declines participation in either EBQI and/or in the evaluation.

### Ethical approval and informed consent

This project was designed as an evaluation in support of VA quality improvement (QI), designated as such by VA Central Office, and approved as such by the IRB at the VA Greater Los Angeles Healthcare System. While participants will not be formally consented, they will also not be mandated to participate and will have the option of not completing surveys and/or interviews. Procedures for secure data transfer (e.g., for interview transcription) and privacy/confidentiality (e.g., de-identified interview data) will be followed.

### Conceptual framework for evaluation

**Fig. 2:**
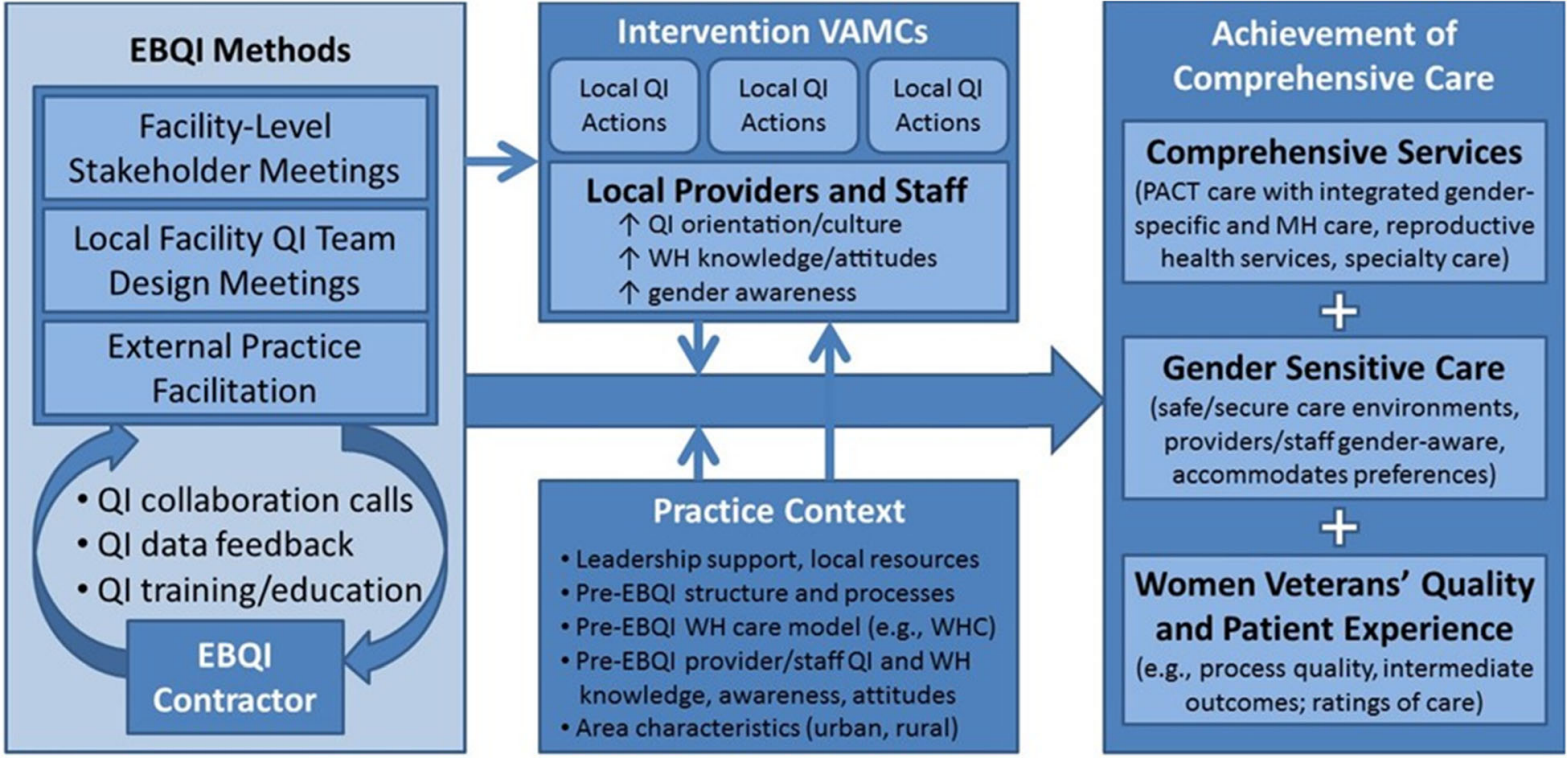
Evidence-Based Quality Improvement (EBQI) conceptual model

We have adapted the conceptual framework from a previous study (Implementation of VA Women’s Health Patient Aligned Care Teams (WH-PACTs)) for the proposed evaluation (Fig. 2) [15]. In this evaluation, a contractor working under technical specifications for EBQI (*far left column*) will (1) convene facility-level stakeholder meetings; (2) facilitate local facility-level QI team design meetings; and (3) provide external practice facilitation through within- and across-facility QI collaboration calls, QI data feedback, and QI training/education. Initial results of EBQI implementation will include local QI actions (e.g., strategic project activities, structured QI proposals, and multilevel key stakeholder review in advance of conduct), and improved provider and staff QI orientation, women’s health knowledge/awareness, and gender awareness (*middle column*, *top*). These actions will occur in the context of each VAMC’s leadership support, local resources, pre-EBQI women’s health care model and staffing, pre-EBQI provider and staff QI and women’s health experience, awareness and attitudes, as well as area characteristics (e.g., urban/rural location) (*middle column*, *bottom*).

Our evaluation activities map directly to this conceptual framework using a formative evaluation framework designed to identify the potential and actual influences on progress and effectiveness of implementation efforts [18]. We will evaluate the EBQI contractor’s implementation of EBQI methods (*first column*) under aim #1 at a *developmental evaluation stage* (e.g., degree of less-than-best practice, determinants of current practice, barriers/facilitators and feasibility/perceived utility). Under aim #2, we will conduct a *progress-focused evaluation* of EBQI effectiveness on achievement of comprehensive care (right column), monitoring impacts and indicators of progress toward goals, with feedback to WHS and the EBQI contractor. Aim #3 will cover two types of evaluation. First, we will conduct an *implementation-focused evaluation* (also known as *process evaluation*) to examine discrepancies between EBQI implementation plans and how the EBQI contractor actually operationalizes them, helping us to identify influences we might otherwise have not considered. This will enable us to describe experiences of sites using EBQI and will consider the context in which facilities participate (12-month key stakeholder interviews will be especially important). Second, we will use results from all of the other evaluation stages to conduct an *interpretive evaluation* (24-month key stakeholder interviews will be key here).

### Evaluation plan

We chose a dynamic wait-listed design for the evaluation to accommodate WHS’s plans for staged EBQI implementation at 21 facilities over 3 years. This randomized “roll-out” implementation design has sound statistical properties, including higher power than traditional wait-listed designs [21], and less vulnerability to external, uncontrolled factors [20]. See Table 1 for an overview of evaluation data sources, samples, and measures described further below.

**Table 1:** Evaluation data sources, samples, and measures

Data sources and samples	Measures
Key stakeholder interviews (baseline, 12 and 24 months follow-up)	
Purposive sample of 130 or more facility-level key stakeholders across the 21 participating sites and corresponding regions	**Baseline interview domains** • Structure and delivery of usual care for women veterans • Barriers and facilitators to achieving delivery of comprehensive women’s health care • Improvements underway in women’s health and/or for women veterans (if any) • Familiarity with performance metrics • Access to metrics by gender • Experience with quality improvement • Local culture • Perceptions of the care environment • Women veteran engagement **12- and 24-month interview domains** • Changes in care for women veterans • Details of completed/in progress QI projects • Perspectives on critical components of EBQI • Anticipated sustainability of local improvements and QI methods
Organizational surveys (annual)	
Key informant organizational surveys, in addition to annual administered WATCH surveys from WHS	Surveys include measures of: • Leadership support • Local resources (e.g., sufficiency of time, personnel, equipment) • Practice structure (e.g., women’s health care model, staff mix, referral arrangements) • Service availability • Care coordination arrangements (within and outside VA) • Ability to engage in QI (e.g., barriers to QI, data access by gender) • Gender-sensitivity of environment (e.g., privacy) • Local challenges (e.g., provider shortages, hiring difficulties, practice chaos) • Facility type (e.g., size, academic affiliation, urban/rural) • EBQI activities
VA provider and staff surveys (annual)	
Census of PC and women’s health providers using Primary Care Management Module data	• EBQI exposure/participation (e.g., awareness, hours spent, local buy-in) • QI orientation/culture (e.g., perceived cooperation among managers/providers/staff, communication effectiveness, culture fostering flexibility, participative decision-making) • Gender sensitivity (e.g., awareness, knowledge, attitudes, self-assessment of women’s health proficiency) • Practice context (e.g., leadership norms, organizational readiness to change, job satisfaction, burnout) • Provider/staff characteristics (e.g., age, gender, race, ethnicity, staff type, clinician type, women’s health provider, proportion of women veterans in panel/clinic, board certification, years in VA)
Administrative data (retrospective data pulls for each year)	
Secondary data on women veteran-specific VA quality of care and patient experience, utilization patterns, and other administrative data	• Quality of care measures from VA performance measures (chart-based and patient survey-based measures by gender), including prevention and chronic disease metrics (e.g., immunizations, cancer screening, diabetes process measures) and patient ratings of access, continuity, and coordination • Utilization measures (e.g., primary care visit rates) • Organizational measures (e.g., facility complexity) • Provider characteristics (e.g., primary care and women’s health provider types, staffing levels)

### Data sources and measures

#### Key stakeholder interviews

Semi-structured qualitative interviews will be conducted at baseline for all sites and at 12 months post-EBQI launch, by group. Interviews will also be conducted with sites in group 1 (the only group for which time permits a second follow-up) at 24 months. We will also interview WHS leaders and EBQI contractor personnel to evaluate leadership and implementation processes. Key stakeholder selection will be adapted based on the QI targets established at initial EBQI site visits (e.g., interview a mental health (mental health) director if the QI project targets mental health). We will seek to re-interview the same key stakeholders from baseline at follow-up but will pursue replacement personnel in the event of turnover and/or position changes over time.

The baseline interview guide includes questions about the *structure and delivery of usual care for women veterans*, *barriers and facilitators* to achieving delivery of comprehensive women’s health care, what (if any) improvements are underway in women’s health and/or for women veterans, *familiarity with performance metrics*, *access to metrics* by gender, *experience with QI*, *local culture*, *perceptions of the care environment*, and *engagement of women veterans in local initiatives* (e.g., Women’s Health Council). The 12- and 24-month interviews will assess any *changes in care for women veterans* (staffing, structure, etc.), details of *completed/in progress QI projects*, perspectives on *critical components of EBQI*, and anticipated *sustainability of local improvements* and QI methods. All key stakeholder interviews will be conducted by telephone, recorded, and professionally transcribed. Transcripts will be reviewed and edited for accuracy.

#### Organizational surveys

We will use key informant organizational surveys at annually among the 21 participating VA facilities, in addition to annual administered WATCH surveys from WHS. For years 2 and 3, we will re-administer the same surveys, adapting selected domains in relation to EBQI targets of participating VAs. We will include measures of *leadership support* [22], *local resources* (e.g., sufficiency of time, personnel, equipment) [23], *practice structure* (e.g., women’s health care model, staff mix, referral arrangements), *service availability* [24], *care coordination arrangements* (within and outside VA), *ability to engage in QI* (e.g., barriers to QI, data access by gender), *gender-sensitivity of environment* (e.g., privacy), *local challenges* (e.g., provider shortages, hiring difficulties, practice chaos) [25, 26], *facility type* (e.g., size, academic affiliation, urban/rural), and *EBQI activities* [17]. We will field surveys through REDCap, a VA-approved web survey vendor.

#### VA clinician/staff surveys

We will use web-based clinician/staff surveys at annually that include measures of *EBQI exposure/participation* (e.g., awareness, hours spent, local buy-in), *QI orientation/culture* (e.g., perceived cooperation among managers/providers/staff, communication effectiveness, culture fostering flexibility, participative decision-making) [27–29], *gender sensitivity* (e.g., awareness, knowledge, attitudes, self-assessment of women’s health proficiency) [30], *practice context* (e.g., leadership norms, organizational readiness to change, job satisfaction, burnout) [31–33], and *provider/staff characteristics* (e.g., age, gender, race, ethnicity, staff type, clinician type, designated women’s health provider, proportion of women veterans in panel/clinic, board certification, years in VA). We will obtain lists of local clinicians and staff by drawing a census from Primary Care Management Module data for each participating facility.

#### VA administrative data

We will pull secondary data on VA quality of care and patient experience for each fiscal year of the evaluation, in addition to utilization patterns and other administrative data on women veterans relevant to the evaluation. Measures will include process measures of *quality for diabetes and cardiovascular disease* (e.g., lipid screening) care and *intermediate outcome measures* (e.g., glycemic and lipid control), access, continuity, coordination, courtesy, and overall satisfaction with VA care. Additional measures include *access* (e.g., average wait time, mental health), *continuity* (% of visits with PACT team providers), *coordination of care* (e.g., emergency room use), *non-face-to-face access* (e.g., telephone visits), *utilization measures* (e.g., outpatient women’s health, mental health, visit rates), and area measures (e.g., urban/rural location, academic affiliation, facility complexity score).

### Analysis plan

#### Qualitative analyses (aims #1 and #3)

Analysis of key stakeholder interviews will initially focus on data consolidation [34] through the use of templated summaries [35] informed by the interview guide, and then organized into matrices to compare and contrast findings across roles, sites, and levels (e.g., facility, Veterans Integrated Service Network (VISN)). In-depth analysis of the key stakeholder interviews will be done using ATLAS.ti, a qualitative data analysis software program that facilitates comparison of data across types and sources. Using a constant comparison analytic approach, the analysis team will develop a top-level codebook and refine it based on emergent themes, particularly as each round of interviews is completed [36, 37]. Analysts will compare and contrast interviews within facility, across facilities, and over time. Consistent with our implementation-focused evaluation in the women’s health-PACT trial, we will explore which women’s health EBQI components are of particular value in improving care and examine clinic and provider characteristics associated with varying levels of EBQI effectiveness and achievement of comprehensive care.

#### Quantitative analyses (aim #2)

We will examine multiple outcome measures as dependent variables: (1) multiple individual measures of comprehensive care achievement, including levels of women’s health service availability (as noted in VHA Handbook 1330.01) [14], integration of and access to gender-specific and mental health care, and other related measures that capture different domains of comprehensiveness; (2) gender-sensitive care delivery, including organizational and provider/staff level measures; and (3) quality of care and patient experience measures. For comprehensive care achievement, we will include as dependent variables the individual measures, and we will also examine approaches to creating an aggregated ordinal score of the individual measures. We will prioritize the final set of dependent variables in consultation with WHS.

The primary regressors of interest will be EBQI exposure (i.e., implementation) and time. We will examine the potential moderating effects of practice context and provider/staff knowledge/attitudes (e.g., determine EBQI effects in high vs. low leadership support sites). We will use multiple linear or logistic regression to evaluate EBQI effectiveness. Where appropriate we will adjust for covariates, account for clustering of patients by site, and mitigate bias due to non-response or loss to follow-up through the use of enrollment/attrition weights. Covariates used for adjustment will include patient factors (e.g., facility case mix, proportion of women veterans seen), provider/staff factors (e.g., designated provider availability), and organizational factors (e.g., resource sufficiency, facility size).

Clustering by site will be accounted for by fitting hierarchical regression models with random intercepts for the sites using Stata 15 [38]. We will evaluate the goodness-of-fit of a given regression model using standard diagnostics (e.g., Mallow’s statistic (*C*_p_)) [39]. To adjust for potential non-response bias and loss to follow-up over time for the provider/staff survey samples, we will apply enrollment weights using available characteristics of eligible providers/staff and attrition or “inverse probability of inclusion” weights estimated using an appropriately specified logistic regression model [40]. We will use multiple imputation methods to replace missing values among covariates [41], with hot-deck methods used for imputation as needed [42]. We will estimate site-level effects using the hierarchical regression models with random intercepts for sites. While our sample of sites [21] is small for the estimation site-level effects, EBQI trials of fewer sites have noted significant effects [43].

### Trial timeframe

The EBQI evaluation will occur from October 2016 through September 2020 (Fig. 1). The EBQI contractor was approved in early 2016, enabling site selection and randomization of seven VA sites in year 1, seven in year 2, and seven in year 3 (not including one extra site per year in case of dropout).

### Trial status

Data collection.

## Discussion

VA efforts to provide access to competent, gender-specific care for women veterans have faced numerous and persistent challenges [1–8]. In response to these challenges, WHS matched VA funding to enable us to conduct a partnered evaluation of their rollout of EBQI as a new strategy for improving quality of women’s health care in consistently low-performing VA facilities. Building on early evidence of EBQI’s promise in activating local teams and leadership around women’s health improvements in care, WHS asked for technical specifications to enable contracting for EBQI. They then asked us to adapt our women’s health PACT EBQI evaluation methods to determine the ways in which EBQI may help low-performing VAs improve quality of care [15]. In our women’s health PACT study, these methods and measures have revealed new information on women veterans’ needs and experiences, elucidated implementation barriers, and helped identify actionable provider/staff attitudes and knowledge gaps [16, 44, 45].

This study may have limitations. For example, the EBQI contractor may not meet contract deliverables on the same schedule as our evaluation. We plan to proceed with the evaluation even if not all contracted for activities occur, enabling us to still address our evaluation aims. Also, low-performing facilities may suffer from leadership gaps, provider and staff burnout, and other structural and management issues that may complicate their engagement in evaluation activities.

Our evaluation activities map directly to a conceptual framework that was originally designed for a cluster randomized trial of EBQI in women’s health primary care [15]. We will use a formative evaluation framework designed to identify the potential and actual influences on progress and effectiveness of implementation efforts [18]. Evaluation results will inform strategies for optimizing future policy deployment and multilevel engagement strategies with the field, while also informing best practice diffusion. The focus on low-performing VAs will offer new insights, as these less-studied facilities may require uniquely concentrated and/or tailored efforts.

## Data Availability

Consents associated with primary data collection for patient and clinician/staff surveys used to evaluate this implementation strategy did not include permission to share data in publicly available repositories. Re-identification is a particular concern in the key stakeholder interviews because of the specific nature of the roles included in a select number of VA networks and VA medical centers, precluding data sharing outside the VA. De-identified administrative datasets may be eligible for future data sharing once national VA guidance on request and distribution processes are provided (in process). Final datasets will be maintained locally until enterprise-level resources become available for long-term storage and access. The analytical datasets and statistical code used in subsequent publications will be retained for a minimum of 7 years, in accordance with VA record retention policy. Any future public release data datasets determined to meet VA guidelines will be maintained so that a VA-approved auditor or the PI could conduct or facilitate validation if needed.

## Abbreviations

CSHIIP: Center for the Study of Healthcare Innovation, Implementation, and Policy
EBQI: Evidence-based quality improvement
HSR&D: Health Services Research & Development
PACT: Patient Aligned Care Teams
PC: Primary care
QI: Quality improvement
VA: U.S. Department of Veterans Affairs or Veterans Health Administration healthcare system
VAMC: VA medical center
VISN: Veterans Integrated Service Network
WATCH: Women’s Assessment Tool for Comprehensive Health
WHS: Women’s Health Services

## Footnotes

1 Patient Aligned Care Teams are the VA’s version of the patient-centered medical home (PCMH) model.
